# Oxygen Adsorption, Absorption and Diffusion in FeCrNi Medium Entropy Alloy: An Ab Initio Study

**DOI:** 10.1002/cphc.202400885

**Published:** 2024-11-20

**Authors:** Farhan Khalid, Meifeng Li, Jing Liu, Hao Zhang

**Affiliations:** ^1^ Department of Chemical and Materials Engineering University of Alberta Edmonton, Alberta T6G, 1H9 Canada

## Abstract

Despite tremendous efforts to understand interstitial diffusion in bulk alloys, a clear understanding of the principal elemental effect on surface interstitial diffusion is still lacking. In this study, a first‐principles approach is employed to study oxygen interstitial diffusion in FeCrNi medium entropy alloy (MEA) based on principal element content at various subsurface sites. Oxygen adsorption energy on surfaces, solution energy at interstitial sites, and activation energy for oxygen permeation are calculated. The adsorption energy for oxygen cohesion to all investigated surfaces was lowest for the sites containing Cr, suggesting a positive effect of Cr in producing a chromium oxide scale. In addition, we have calculated the contribution of the principal element to the stability of the interstitial sites and the activation energy to diffuse between them. This work provides insights into the formation of chromium scaling based on oxygen adsorption and permeation, with potential implications in the design of oxidation‐resistant surfaces for high‐temperature applications.

## Graphical abstract

Oxidization and formation of passive layer in FeCrNi medium entropy alloy are not clear at the atomic level. Here, using density functional theory, we investigate the adsorption and diffusion of oxygen atoms on different surfaces. The presence of Cr at the surface or subsurface is the most important factor for adsorption energy and the permeation barrier.

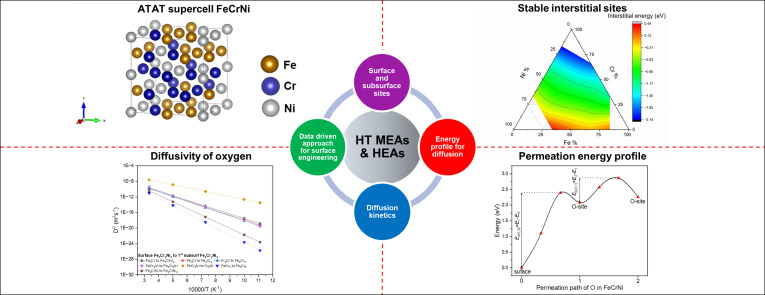

## Introduction

Fe−Cr‐Ni series stainless steels have good corrosion resistance and superior mechanical properties, making them useful as a structural material for various applications, such as aerospace and nuclear power generation.[^1^, ^2^] Stainless steel series have a higher Cr content (around 11–23 %) since Cr promotes the formation of protective oxide film Cr_2_O_3_ on the surface and prevents pitting corrosion.^[3]^ Further corrosion resistance of steel alloys can be improved by adding more Cr; however, when Cr content reaches above 25 wt %, a harmful sigma (σ) phase is formed, leading to a decrease in corrosion resistance.^[4]^ Hence, it is hard to achieve single‐phase traditional alloys with higher Cr content that can be suitable for high‐temperature (HT) applications.

Multi‐principal element alloys, including medium entropy alloys (MEAs) and high entropy alloys (HEAs), have greatly attracted material designers for better alloy design.[^5^, ^6^, ^7^, ^8^, ^9^] As MEAs and HEAs are composed of multiple principal elements in equimolar or near‐equimolar ratios, the high mixing entropy enables the alloys to form simple solid solutions rather than complex intermetallic compounds. These simple solid solutions are face‐centered cubic (FCC), body‐centered cubic (BCC), and close‐packed hexagonal (HCP) structures.[^7^, ^10^, ^11^, ^12^] The limit of Cr content in stainless steels can be exceeded using medium and high entropy alloys, avoiding the formation of σ phase and increasing corrosion resistance.

FeCrNi MEA (Cr~33 at %) having single‐phase FCC microstructure has been prepared using powder metallurgy.^[13]^ This alloy has impressive mechanical properties with an ultimate tensile strength of ~1.03 GPa and ductility of ~54 %, which is significantly better than 316 L stainless steel (UTS~485 MPa and ductility ~40 %). Furthermore, as compared to 316 L stainless steel, FeCrNi MEA exhibits superior corrosion resistance, as evidenced by a higher positive breakdown potential and lower current density in 3.5 wt % NaCl solution.^[14]^ The study conducted by Sun et al. identified that a single BCC phase was observed at temperatures below 900 °C but disappeared at higher temperatures to become the FCC phase.^[15]^ Oxygen has higher diffusivity in the BCC phase compared to the FCC phase of FeCrNi MEA,^[15]^ but as the BCC phase is absent above 900 °C, this study only investigates FCC phase surface diffusion. Theoretical studies performed before have examined bulk diffusion in Fe−Cr, Fe−Ni, and FeCrNi systems.[^15^, ^16^, ^17^, ^18^] Since these studies only focus on bulk diffusion, they do not reveal the initial point of oxygen adsorption on the surface and the permeation pathways from the surface to the bulk.

The studies conducted previously examined the overall corrosion in MEAs and HEAs. The general impact of the principal element on oxidation and corrosion kinetics is evident. However, a deeper dive into the principal elements’ compositional and configurational impact on HT oxidation of the FeCrNi system has not been undertaken. In this work, we employ density functional theory (DFT) calculations to investigate the oxidation mechanism in FeCrNi MEA based on the Special Quasi‐random Structures (SQS) generated supercells for the FCC phase. Oxidation starts at the surface for which surface adsorption for all different combinations of sites is considered for (001), (110), and (111) low Miller index planes. Then, the absorption and permeation pathway of oxygen to the subsurface is calculated. This computational approach enhanced our understanding of the oxidation mechanism at the surface by considering all possible combinations of initial and final sites. This information is critical for understanding and predicting the behavior of alloys under operating conditions, which will ultimately aid in the development of more robust and oxidation‐resistant materials by surface engineering. To our knowledge, this is the first comprehensive density functional theory study of the energetics of oxygen and its diffusion in the near‐surface regions of the FeCrNi system that considers the combinational effect of principal elements.

## Methods

### First Principles Calculation

Our first‐principles calculations are based on density functional theory^[19]^ and performed using the Vienna Ab‐Initio Simulation Package (VASP).[^20^, ^21^] Ion electron interactions were evaluated by the projector augmented‐wave (PAW) method.[^22^, ^23^] The electron exchange and correlation were described within the generalized gradient approximation (GGA) by Perdew‐Burke‐Ernzerhof (PBE).^[24]^ All the calculations were performed with the spin‐polarized and a kinetic energy cutoff of 450 eV, which was found to be sufficient for convergence of the adsorption and interstitial energies. Structural relaxations were performed until the maximum force on any moveable atom in any direction was less than 0.01 eV/Å. The total energy change and the band structure energy change between the two steps were smaller than 10^−6^ eV. A 3×3×3 Monkhorst‐Pack k‐point meshes is used for the Brillouin‐zone interactions, and maximum symmetry is applied to reduce the number of k‐points in the calculations.

Complications arise when DFT is used to generate structures of randomly mixed solid solutions, e. g., multicomponent MEAs. One strategy is to construct large supercells to obtain a multi‐component structure, where the constituent atoms randomly decorate the host lattices. However, this can be computationally expensive. Another approach is to use the Special Quasirandom Structures developed by Zunger et al.[^25^, ^26^] with small‐unit‐cell periodic structures that can mimic the most relevant information, such as the pair and multisite correlation function of the alloy. The supercells of various solid solution phases in the present examined MEA have been built using the mcsqs code (using a Monte Carlo algorithm to find an SQS implemented in the Atom‐Theoretic Automated Toolkit (ATAT)).^[26]^ Using this ATAT, a 2×2×2 FCC supercell (32 atoms) of equimolar FeCrNi was built with lattice constants *a*=3.515 Å, *b*=3.510 Å and *c*=3.522 Å.^[27]^

A *p*(2×2) surface cell and a k‐mesh of 3×3×1 were used to study the oxygen adsorption process on FeCrNi (001), (110), and (110) and permeation processes through the FeCrNi (001). A 20 Å thick vacuum layer was employed to study the O adsorption and permeation. The bottom three atom layers were fixed at their bulk positions, while the other layers were geometrically relaxed.

We employed the climbing image nudged elastic band (CI‐NEB) method to estimate the transition states, diffusion barriers, and the permeation process through FeCrNi (001).[^28^, ^29^] The CI‐NEB method is reliable in finding saddle point and minimum energy pathways with known initial and final states.

### Computational Details of Surface Adsorption

It is well known that the oxidation of metallic alloys initiates from the adsorption of oxygen on their free surfaces. Therefore, we first calculated the adsorption energies of oxygen atoms on different surface orientations and surface compositions of FeCrNi. The atomic positions of the free layers were allowed to relax to reach their equilibrium positions before interacting with oxygen atoms. Thereafter, the oxygen atom was placed on the top of the different sites (on the relaxed free surface) at 1.80 Å from the surface plane to calculate the total formation energy. The internal energy of the isolated oxygen atom, $E_{O}$ , was calculated as half of the energy of the O_2_ molecule, $E_{O_{2}}$ . The adsorption energy was then calculated using Equation 1, where $E_{ads}$ is the adsorption energy, $E_{total}\;$ is the energy of the model with oxygen, and $E_{slab}$ is the total energy of the model without oxygen.

(1) $$E_{ads}=E_{total}-\left(E_{slab}+{\frac{1}{2}E}_{O_{2}}\right)$$

The purpose of this calculation is to investigate the effect of each principal element on the initiation point of surface oxidation. To cover all possible compositional combinations, multichoice binomial equation Equation 2 is employed to calculate unique four‐fold and three‐fold adsorption sites,^[30]^ where *n* is the number of metal atoms at the adsorption site and *r* is the number of principal elements. Specifically, for the four‐fold adsorption site, *n* is four, and for the three‐fold adsorption site, *n* is 3; *r* is 3 for both. Hence, DFT calculations of 15 cases for (001), 15 cases for (110), and 10 cases for (111) were carried out.

(2) nr=n+r-1!n!r-1!

Surface energy, $\sigma_{hkl}$ , for any orientation (*h k l*) can be calculated using Equation 3. $\sigma^{relax}$ defines the energy of the system with relaxed surface (free layers), and $\sigma^{unrelax}$ defines the energy of the system with unrelaxed (fixed layers). $\sigma^{unrelax}$ is further calculated using Equation 4 where $E_{Surf-fixed}$ is the energy of the system with a fixed surface, $N_{atoms}$ is the ratio of atoms in the surface slab and bulk and $E_{bulk}$ is the energy of bulk system.

(3) $$\sigma_{hkl}=\sigma^{relax}+\sigma^{unrelax}$$

(4) $$\sigma^{unrelax}=E_{Surf-fixed}-N_{atoms}\times E_{bulk}$$

### Surface Permeation and Arrhenius Diffusion Constant

Diffusion of interstitial oxygen in the bulk material is governed by the classical Arrhenius diffusion constant expression $D^{o}=D_{o}exp(\frac{-E_{b}}{k_{B}T}$ ), where $E_{b}$ is the activation energy, i. e., diffusion barrier, $k_{B}$ is the Boltzmann constant and T is the absolute temperature. Based on harmonic Transition State Theory (hTST) in the classical limit and the random‐walk model of interstitial diffusion in an FCC lattice, the pre‐exponential factor $D_{o}$ is calculated using the frequency of the initial minimum state and transition state. In $D_{o}=\frac{n}{6}l^{2}v_{o}$ , n is the geometrical factor for the number of equivalent jump paths (4 for O in the O‐site of FCC), l is site to site jump length and $v_{o}$ includes the frequency term.[^31^, ^32^] The value of 1/6 means that the diffusion is equal to probability in the six directions (±x, ±y, and ±z). In Equation 5 for $v_{o}$ , N are the number of atoms and $v_{i}^{I}$ and $v_{i}^{T}$ are the real normal mode frequencies at the initial and transition states.

(5) $$v_{o}=\frac{\prod_{i}^{3N}v_{i}^{I}}{\prod_{i}^{3N-1}v_{i}^{T}}$$

To check the thermodynamic feasibility of different permeation pathways, we can think of them in terms of the energy of the starting point, the transition (saddle point), and the endpoint. Equation 6 defines *ΔH*, the driving force, as the change in energy between the final site *E_final_* and the initial site *E_initial_*.

(6) $$\Delta H=E_{final}-E_{initial}$$

To calculate the activation energy associated with a permeation pathway, Equation 7 defines *E_b_* as the difference of energy between the transition site energy *E_transition_*, and the initial site energy *E_initial_*.

(7) $$E_{b}=E_{transition}-E_{initial}$$

Lastly, *E_I_* defines the solution energy of oxygen at the octahedral site, which is calculated using Equation 8, where *E_total_* is the total energy of the system with the interstitial, $E_{slab}$ is the total energy of the system without the interstitial, and $\mu_{i}$ is approximated as the total energy of an individual atom of the interstitial that is inserted in the system.^[33]^

(8) $$E_{I}=E_{total}-E_{slab}-\mu_{i}$$

## Results and Discussion

### Surface Adsorption of Oxygen

**Figure 1:**
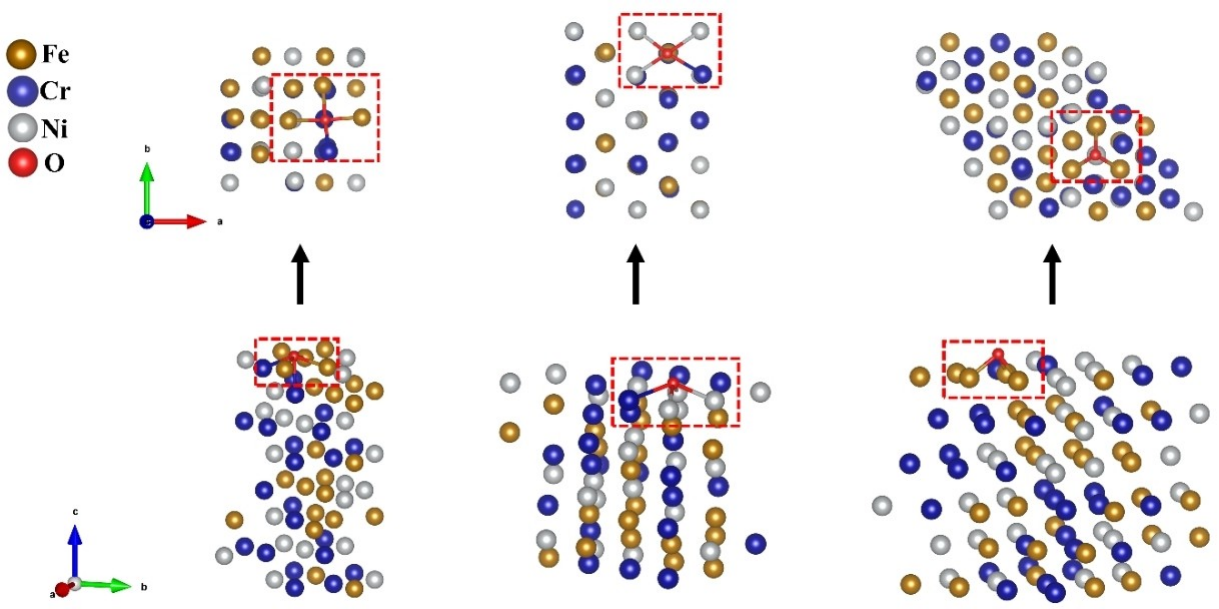
Top and isometric views of FeCrNi supercells adsorbing oxygen atoms on different oriented surfaces. The top view and the isometric view of: (a) (001) (b) (110) show a fourfold hollow site, and (c) (111) show a three‐fold hollow site. An oxygen atom is positioned in the middle at the hollow site.

Calculated adsorption energies for all the surface cases are compared and analyzed. It is found that oxygen atom always prefers to be adsorbed at a four‐fold hollow site on (001) and (110) surfaces, whereas for (111) surface they prefer the three‐fold hollow sites as shown in Figure 1.

**Figure 2:**
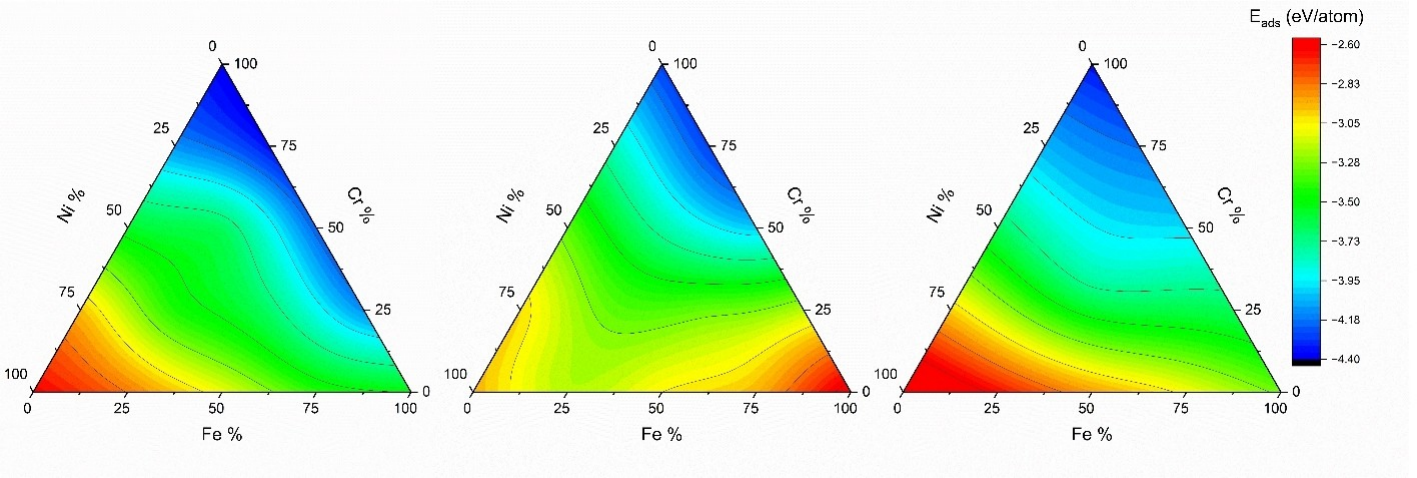
The adsorption energy of oxygen atom at the adsorption site surface versus % composition of the principle elements at the adsorption site. (a) four‐fold adsorption site energy for (001), (b) four‐fold adsorption site energy for (110), and (c) three‐fold adsorption site for (111).

Regardless of surface orientation, the calculated results showed that adsorption energy mainly depends on the neighboring metal atoms around the oxygen atom at the hollow sites on the free surface. As there are three different principal elements in FeCrNi MEA, there exist many combinations of the adsorption sites. In this present work, we have used Equation 2 to determine the combinations of these adsorption sites for the three surface orientations before starting surface adsorption energy calculations. The data for oxygen adsorption on (001), (110), and (111) surfaces is shown in Figure 2 where a ternary plot shows the relationship of principle element composition at the adsorption site on adsorption energy *E*_*ad*s_. Figure 2(a) shows the adsorption energy of the oxygen atom on the (001) surface at the four‐fold hollow site as a function of the changing composition of Fe, Cr, and Ni. As it is evident, the adsorption energy of oxygen on (001) surface of FeCrNi MEA alloy decreased with increasing % of Cr while it increased with increasing % of Ni. The highest adsorption energy is for a site containing 100 % Ni (Ni_4_−2.61 eV/atom). The lowest adsorption energy is for a site containing 100 % Cr (Cr_4_−4.35 eV/atom). While Cr and Ni have a strong correlation to the adsorption energy of oxygen, Fe does not show any strong trend for the adsorption of oxygen atoms.

Figure 2(b) highlights the adsorption energy trends for oxygen on the (110) surface of FeCrNi MEA. The highest adsorption energy for this surface orientation is attributed to the four‐fold site containing 100 % Fe (Fe_4_−2.53 eV/atom), while the lowest adsorption energy was observed when all the neighboring atoms contained 100 % Cr (Cr_4_ -4.37 eV/atom). It is noticed that oxygen adsorption energies at the Fe_4_ site on (001) and (110) surfaces are quite distinct. For (110) surface, at Fe_4_ (four‐fold hollow site) oxygen atom adsorbs further from the surface at 2.08 Å away from a Ni atom (vertically) below it in the 1^st^ subsurface. However, on (001) surface it adsorbs closer to the surface 1.86 Å away from a Ni atom (vertically) below it in the 1^st^ subsurface. This can be the reason for stronger adsorption attributed to the sites containing Fe on (001). Another reason can be the diagonal spacing between oxygen and the surface metal atoms at the four‐fold hollow site of the (110) surface is greater than that of the (001) surface, as can be visualized in Figure 1(a–b). The effect of Cr on oxygen adsorption on (110) and (001) surfaces exhibits a similar trend, i. e., with the increase in Cr atom in the four‐fold site, the adsorption energy of oxygen decreases. However, the effect of Fe is quite different. For the (110) surface, the presence of Fe in the adsorption site plays the same role as the presence of Ni in the (001) surface, causing the adsorption energy to increase.

The adsorption energy of oxygen on the (111) surface of FeCrNi MEA follows the same general trend as the (001) surface in Figure 2(c). The highest adsorption energy was observed when oxygen was surrounded at the three‐fold hollow site by 100 % Ni (Ni_3_−2.35 eV/atom), and the lowest adsorption energy is associated with oxygen being surrounded by all 100 % Cr (Cr_3_ <M->4.35 eV/atom). The influence of Fe atoms on the adsorption energy lies somewhere in between that of Cr and Ni. A previous study by Hong et al. on Al_0.3_CoCrCuFeNi HEA estimated the lowest energy of adsorption for sites containing Cr, Cr_4_ for (001) and (110) facets, and Cr_3_ for (111) facets.^[34]^ Furthermore, this confirms that oxygen's affinity for any alloy containing Fe−Cr‐Ni follows the trend Cr>Fe>Ni, and adsorption energy depends on the principal element present at the adsorption site and not the overall composition of the alloy. (Detailed results of adsorption energy are shown in Figure S.1 in Supplementary Information (SI)).

In our work, we have calculated the average surface energy to follow the trend $\sigma_{111}<\sigma_{001}<\sigma_{110}$ , with (111) surface being the most stable and probable. However, the average adsorption energy trend of these surfaces shows $E_{ads}^{001}<E_{ads}^{111}<E_{ads}^{110}$ , with (001) surface having the lowest oxygen adsorption energy in Table 1. Hence, next oxygen permeation and diffusion will be investigated on the (001) surface.

**Table 1:** Average Surface energy and Average adsorption energy of the orientations.

$\sigma_{hkl}$	avg Surface energy (eV)	avg E_ads_ (eV/atom)
$\sigma_{001}$	1.581±0.071	−3.595
$\sigma_{110}$	1.639±0.015	−3.400
$\sigma_{111}$	1.553±0.004	−3.453

Furthermore, the presence of Cr on the surface of the alloy lowers the adsorption energy for all the surface orientations simulated. The improvement in the corrosion and oxidation resistance of the alloy is attributed to the formation of Cr_2_O_3_ on the surface,^[35]^ where Cr sacrifices itself to protect the alloy and hinders oxygen diffusion in the bulk metal. Moreover, a higher Cr content on the surface makes it harder for oxygen to diffuse to the subsurface. This will be discussed in the following sections.

### Surface to 1^st^ Subsurface Diffusion

Oxygen diffusion into the subsurface is interstitial diffusion associated with a final stable site. In FCC lattice there are two types of common interstitial sites, i. e., Tetrahedral (T‐site) and Octahedral (O‐site).^[35]^ For FeCrNi MEA, we tested all the possible T‐sites and O‐sites and found oxygen atom is stable at the O‐site. When an oxygen atom was inserted in the T‐site, it was metastable and moved outwards from the subsurface to the surface. The large coordination number of the O‐site can be the reason for its stability, and another reason can be the high Cr content on the surface attracting the oxygen to it from the subsurface, making the T‐site metastable. For each surface, we chose the diffusion starting point as the site with the lowest adsorption energy. The coordination number of oxygen at the O‐site is 6, and the octahedral site can be made up of different compositions of Fe−Cr‐Ni. In our work, for ease of understanding, the composition of an O‐site will be represented as ${Fe}_{x}{Cr}_{y}{Ni}_{Z}$ (e. g., an O‐site containing 2 Fe, 2 Cr, and 2 Ni atoms is denoted as Fe_2_Cr_2_Ni_2_).

**Figure 3:**
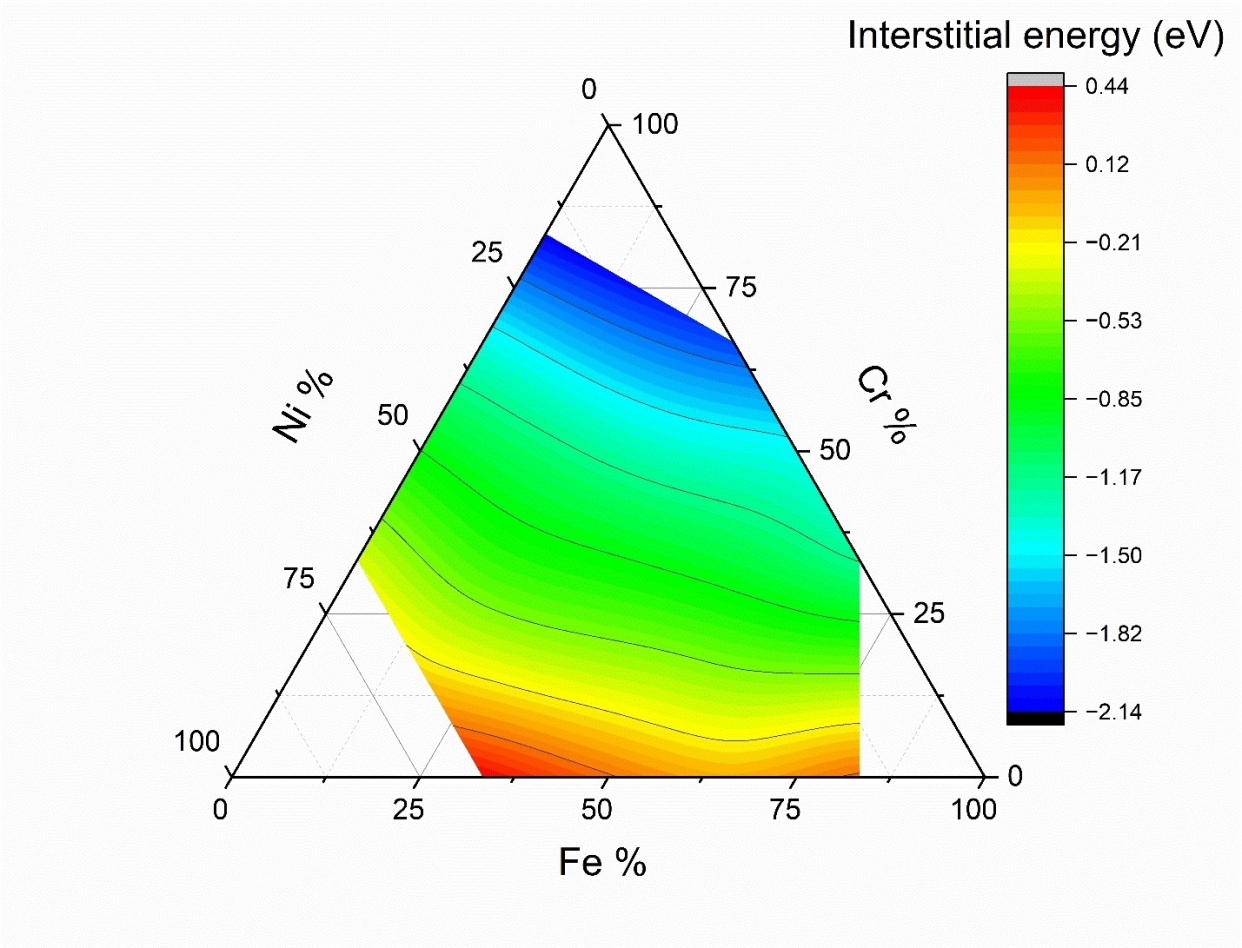
Calculated interstitial energy of oxygen atom at various octahedral sites in bulk FeCrNi MEA.

After oxygen is adsorbed to the four‐fold hollow site on (001) surface, it has 4 adjacent O‐sites to diffuse to in the 1^st^ subsurface. Oxygen atom will prefer to diffuse to the O‐site which has the lowest *ΔH* and *E*_*b*._ Figure 3 illustrates the interstitial energy *E_I_* as a function of the changing composition of Fe, Cr, and Ni at the stable O‐sites. The interstitial energy *E_I_* of oxygen at the O‐site follows the same trend as its adsorption energy, with the increasing presence of Cr, the interstitial site becomes more stable, and with the increasing presence of Ni or Fe, the site becomes less stable. (Detailed results of interstitial energy are shown in Table S.1 in Supplementary Information (SI)).

**Figure 4:**
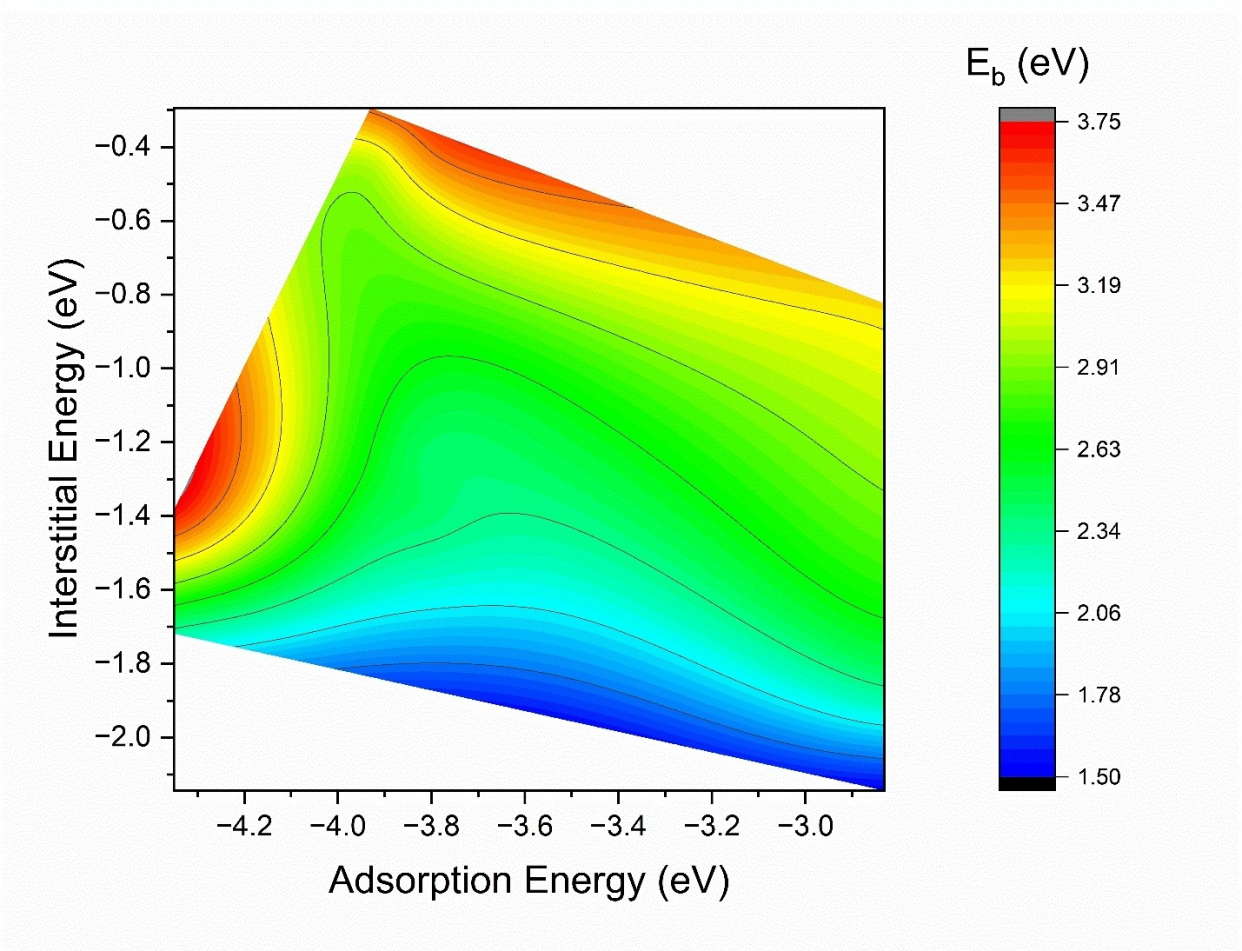
Variation of *E_b_* for various (001) *E_ads_* surface adsorption energy and *E_I_* interstitial energy at the 1^st^ subsurface.

Figure 4 describes how the energy *E_b_* for diffusion from the (001) surface to the 1^st^ subsurface varies as a function of the adsorption energy at the surface and interstitial energy at the 1^st^ subsurface change. Oxygen prefers to be adsorbed to Cr‐containing sites on the surface, however, this makes it harder for oxygen to diffuse into the 1^st^ subsurface, as shown in Figure 4. Generally speaking, if the subsurface interstitial energy is low (e. g., *E_I_* <−1.7 eV), then the energy barrier for diffusion is also relatively low. Sites containing Cr have the lowest adsorption and interstitial energies, making it harder for oxygen to diffuse as compared to sites contacting Fe or Ni, as they have higher adsorption and interstitial energy. Lower adsorption energy and lower interstitial energy generally mean that both sites are very stable (large Cr content), hence *E_b_* would be higher as can be seen by the red and yellow regions of the figure. The highest energy barrier *E_b_* 3.75 eV is associated with oxygen diffusing from FeCr_3_ to Fe_3_Cr_3_ O‐site, while the lowest *E_b_* 1.50 eV is associated with FeCr_2_Ni to Cr_5_Ni O‐site. The presence of Cr on the surface makes it easier for oxygen to be adsorbed, while the presence of Cr in the subsurface makes it easier for oxygen to diffuse.

**Figure 5:**
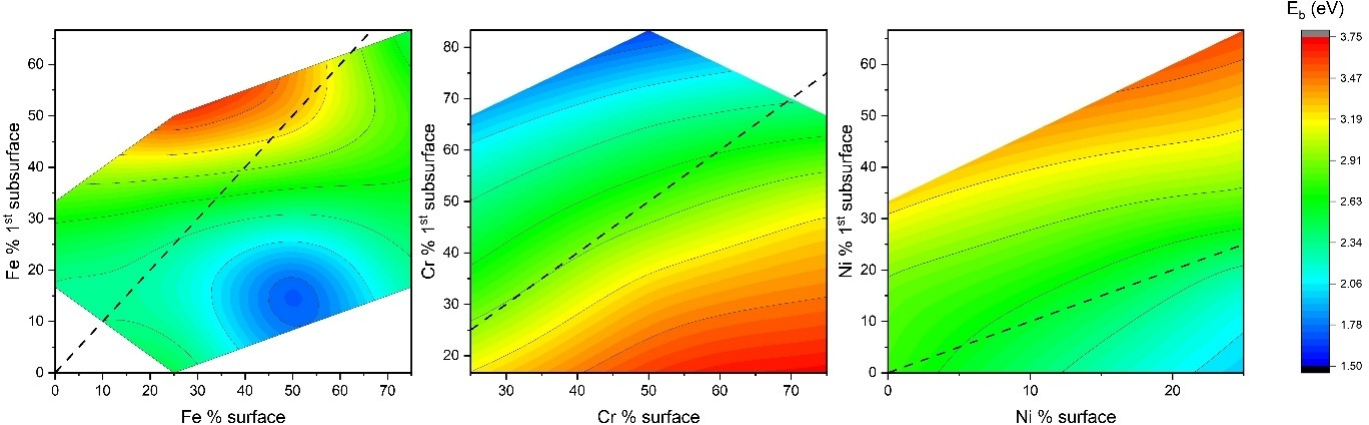
Influence of principal element on the (001) surface to 1^st^ subsurface diffusion barrier *E_b_*: (a) Fe % on the surface vs. Fe % in the 1^st^ subsurface, (b) Cr % on the surface vs. Cr % in the 1^st^ subsurface and (c) Ni % in the surface vs. Ni % in the 1^st^ subsurface. The dotted black line indicates the percentage composition of the element does not change from the surface to the subsurface.

Figure 5 describes how the *E_b_* is influenced by the change in the principal element at the surface and 1^st^ subsurface. The black dotted line indicates no change in the amount of principal element before and after the diffusion. It can be seen from Figure 5(a) that if there is more Fe on the surface compared to the subsurface, *E_b_* is lower, while in Figure 5(b) if there is more Cr on the surface compared to the subsurface, *E_b_* is higher. Similarly, in Figure 5(c) more Ni on the surface compared to the subsurface makes *E_b_* lower, which is similar to the effect of Fe. However, for Ni the *E_b_* magnitude is lower compared to Fe. The average *E_b_* for all the calculated cases was 2.69 eV from surface to 1^st^ subsurface and 0.21 eV from 1^st^ subsurface to surface for the pathways investigated. This suggests that oxygen in FeCrNi MEA prefers to remain on the surface rather than diffuse in the subsurface. (Detailed results of activation energy for surface to 1^st^ subsurface Table S.2 in SI).

### 1^st^ Subsurface to 2^nd^ Subsurface Diffusion

**Figure 6:**
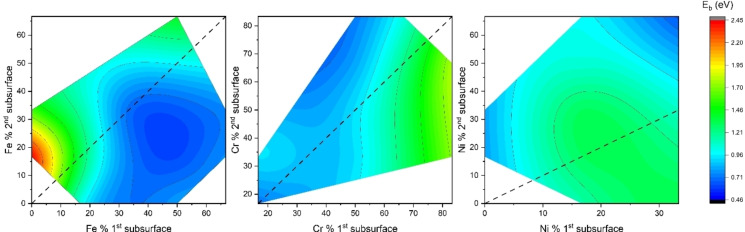
Influence of principal element on (001) 1^st^ subsurface to 2^nd^ subsurface diffusion *E_b_*: (a) Fe % in the 1^st^ subsurface vs. Fe % in the 2^nd^ subsurface, (b) Cr % in the 1^st^ subsurface vs. Cr % in the 2^nd^ subsurface and (c) Ni % in the 1^st^ subsurface vs. Ni % in the 2^nd^ subsurface. The dotted black line indicates the percentage composition of the element does not change from the surface to the subsurface.

The diffusion activation energy *E_b_* of oxygen between the 1^st^ and 2^nd^ subsurface (see Figure 6) is generally smaller as compared to that between the surface and 1^st^ subsurface depicted in Figure 5. Oxygen is more stable in the 2^nd^ subsurface than the 1^st^ subsurface for the cases studied. *ΔH* for some of the investigated pathways was negative, which was not observed for surface to 1^st^ subsurface diffusion. This highlights that for some pathways the diffusion between 1^st^ and 2^nd^ has a positive driving force and diffusion between these two surfaces will be preferred.

Similar to the 1^st^ subsurface, the most stable site for oxygen in the 2^nd^ subsurface is the O‐site, and oxygen follows the same pathway of O‐site to O‐site diffusion. The amount of chromium determines which O‐site in the 1^st^ subsurface or 2^nd^ subsurface is more stable, and hence the *E_b_* for diffusion. The presence of Cr in the 1^st^ subsurface makes it harder for oxygen to diffuse, while the presence of Cr in the 2^nd^ subsurface aids in the diffusion of oxygen. Figure 6 describes the *E_b_* influenced by the change in the principal element at the 1^st^ subsurface and 2^nd^ subsurface. The black dotted line indicates no change in the amount of principal element before and after the diffusion. In Figure 6(b), the area under the dotted line shows a higher *E_b_* compared to the area above the line when Cr content in the 1^st^ subsurface is higher below the dotted line. This explains that Cr positioning in the 1^st^ subsurface or the 2^nd^ subsurface also determines the diffusion kinetics.

The presence of Fe shown in Figure 6(a) has the opposite effect compared to Cr, where the area under the dotted line has a lower *E_b_* as compared to the area above the dotted line. Ni has a varying effect in Figure 6(c), showing an opposite trend when compared to surface to 1^st^ subsurface Figure 5(c), i. e., the region below the line has higher *E_b_* as compared to the region above the line. However, some regions above the line do have a slightly high *E_b_* of 1.46 eV, which is different from surface to 1^st^ subsurface. The lowest *E_b_* 0.46 eV reported is for the case of Fe_3_CrNi_2_ (O‐site 1^st^ subsurface) to FeCrNi_4_ (O‐site 2^nd^ subsurface), as Fe decreases and Ni increases, the *E_b_* decreases. The highest *E_b_* 2.45 eV is associated with Cr_5_Ni (O‐site 1^st^ subsurface) to FeCr_4_Ni (O‐site 2^nd^ subsurface), showing that a decrease in Cr and an increase in Fe lead to a larger *E_b_*.

The *E_b_* trend for surface to 1^st^ subsurface and 1^st^ subsurface to 2^nd^ subsurface follows the same general rule reported by Xiong et al. In their paper, they determined hydrogen permittivity in Fe to have a very high *ΔH* and *E_b_* for surface to 1^st^ subsurface diffusion, which decreases as the hydrogen further diffuses to deeper sublayers.^[36]^ Oxygen in FeCrNi MEA exhibits the same high *ΔH* and *E_b_* for initial oxygen permeation which further decreases for deeper sublayers. Diffusion of oxygen is mainly governed by the presence of Cr and depends upon where the Cr is present (start site or end site). On the other hand, Fe has the opposite effect of Cr, and Ni's effect lies somewhere in between that of Cr and Fe. (Detailed results of activation energy for 1^st^ subsurface to 2^nd^ subsurface are shown in Table S.3 in SI)

### Diffusivity of Oxygen in FeCrNi MEA

**Figure 7:**
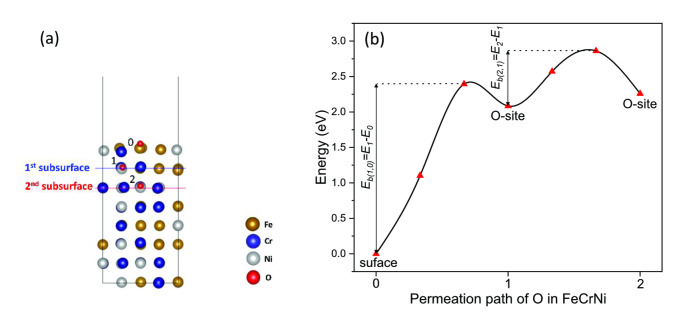
(a) The permeation pathway of O in (001) surface from stable four‐fold adsorption site (0) to O‐site in 1^st^ subsurface (1) and further to O‐site in 2^nd^ subsurface (2) and (b) Energy profile for adsorbed O and its permeation pathway to the 1^st^ and 2^nd^ subsurface and energy barrier of O diffusion in (001) surface. *E*_*b(1,0)*_ and *E*_*b(2,1)*_ are the energy barrier from the surface to 1^st^ subsurface and 1^st^ subsurface to 2^nd^ subsurface, respectively.

Figure 7 illustrates the interstitial oxygen diffusion mechanism in FeCrNi MEA, starting with surface adsorption (0), then with the inward diffusion of oxygen to the 1^st^ subsurface O‐site (1) and then deeper to the 2^nd^ subsurface O‐site (2). Figure 7(b) shows the general energy barrier curve along the permeation pathway. For surface to 1^st^ subsurface diffusion, both *ΔH* and *E_b_* are generally higher (see Figure 5 & Figure 6) as oxygen is diffusing in from the vacuum to the lattice. The *ΔH* and *E_b_* are smaller for 1^st^ subsurface to 2^nd^ subsurface diffusion as oxygen is already in the lattice.

**Figure 8:**
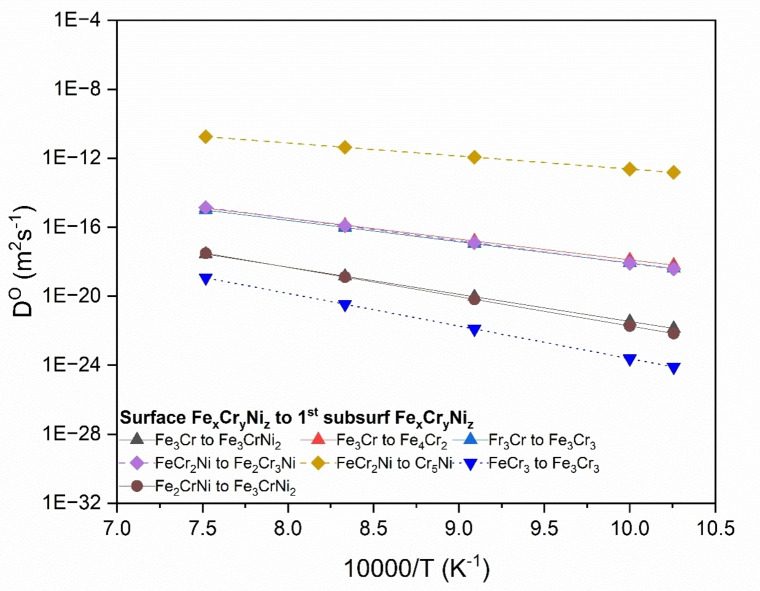
Arrhenius plots of diffusivity values calculated from (001) surface four‐fold adsorption site (Fe_x_Cr_y_Ni_z_) to 1^st^ subsurface O‐site (Fe_x_Cr_y_Ni_z_).

Figure 8 shows the calculated diffusion coefficient of oxygen *D* ^*O*^ in the FCC phase of FeCrNi from the surface to the 1^st^ subsurface. *D* ^*O*^ estimation includes calculating $E_{b}$ , which is the energy barrier (activation energy) associated with surface to 1^st^ subsurface interstitial diffusion, and calculating pre‐exponential factor, $D_{o}$ , which involves estimation of the frequency term $v_{o}$ (see Equation 5). The initial oxidation process is controlled by the inward diffusion of oxygen, which is true for many alloys.^[15]^ As discussed earlier in terms of adsorption energy and energy barriers of surface to 1^st^ subsurface diffusion, the presence of certain elements greatly affects diffusion kinetics. The largest *D* ^*O*^ is associated with diffusion from the four‐fold adsorption site FeCr_2_Ni to Cr_5_Ni. This is because the end O‐site has a very large amount of Cr as compared to the initial site, and Fe reduces to zero and Ni stays the same, which enables O at the end O‐site to have a very low (stable) interstitial energy. The lowest *D* ^*O*^ is associated with O diffusion from the FeCr_3_ four‐fold adsorption site to the Fe_3_Cr_3_ O‐site. For this case, there is a big change in Cr percentage between the two sites, with Cr content being lower at the end O‐site, whereas Fe increases at the end site. The end O‐site has a higher (less stable) interstitial energy due to less Cr and more Fe, making it easier for oxygen to stay at the surface. Oxygen prefers to remain on the surface due to higher Cr content, causing this diffusion pathway to have a higher *E_b_*. From diffusivity curves we can see how *D* ^*O*^ varies as principal elements change.

**Figure 9:**
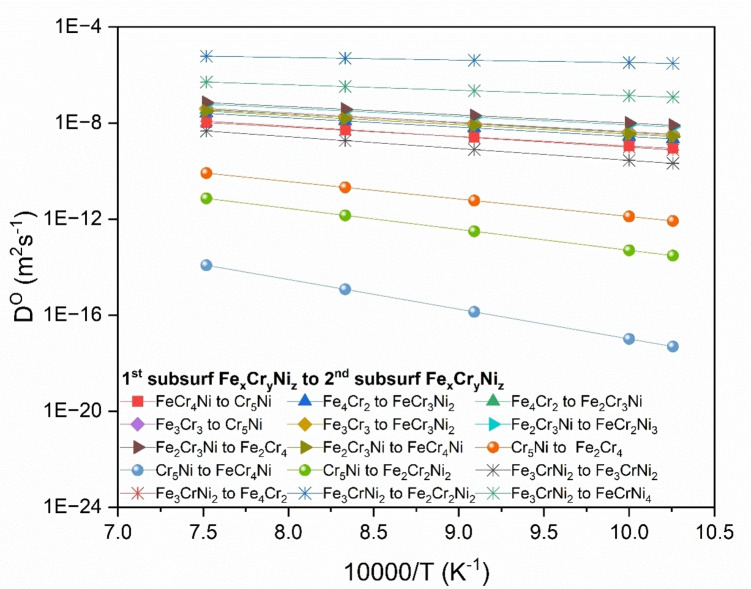
Arrhenius plots of diffusivity values of oxygen calculated from (001) 1^st^ subsurface O‐site (Fe_x_Cr_y_Ni_z_) to 2^nd^ subsurface O‐site (Fe_x_Cr_y_Ni_z_).

In general, *D* ^*O*^ for 1^st^ subsurface to 2^nd^ subsurface is larger for all the cases as compared to the diffusivity from the surface to 1^st^ subsurface as shown in Figure 9. This is due to the relatively smaller *E_b_* and in some cases a negative *ΔH*. The largest *D* ^O^ is associated with oxygen diffusing from the Fe_3_CrNi_2_ O‐site at 1^st^ subsurface to the Fe_2_Cr_2_Ni_2_ O‐site at 2^nd^ subsurface. Between the two O‐sites, Cr content increases while Fe decreases slightly. This small change in the percentages of Cr and Fe results in a smaller *E_b_* 0.21 eV, since the end O‐site has more Cr attracting oxygen. Furthermore, it can be explained by the transition state of this O‐site to O‐site diffusion, as the transition site T‐site contains 2 Cr and 2 Fe atoms. The higher concentration of Cr in the transition site lowers the transition site energy *E_transition_*, resulting in a lower *E_b_*. The difference in interstitial energy of oxygen between 1^st^ subsurface and 2^nd^ subsurface is smaller than that in the surface and 1^st^ subsurface. Hence, the elemental effect of any principal element especially Cr for 1^st^ subsurface to 2^nd^ subsurface diffusion is smaller as compared to the surface to 1^st^ subsurface. This energy trend again is confirmed when we account for the case with the lowest *D* ^*O*^, Cr_5_Ni O‐site to FeCr_4_Ni O‐site *E_b_* 2.45 eV, i. e., Cr decreases at the end site while Fe increases. The transition site T‐site for this pathway is associated with having lower Cr content leading to higher transition site energy *E_transition_*, hence resulting in a higher *E_b_* and a larger change in *D* ^*O*^.

### PDOS of Oxygen Permittivity

**Figure 10:**
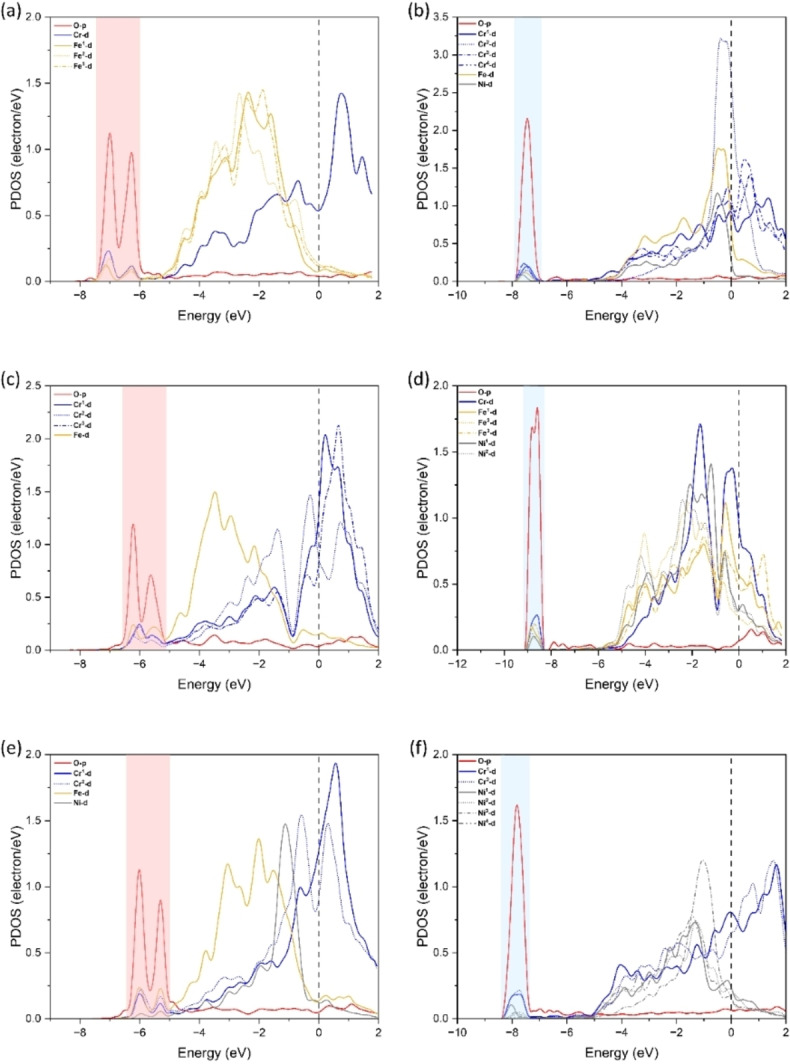
Projected density of states (PDOS) of configurations of FeCrNi (001) surface with one oxygen atom: (a‐b) O at Fe_3_Cr & FeCr_4_Ni, (c‐d) O at FeCr_3_ & Fe_3_CrNi_2_ and (e‐f) O at FeCr_2_Ni & Cr_2_Ni_4_.

**Figure 11:**
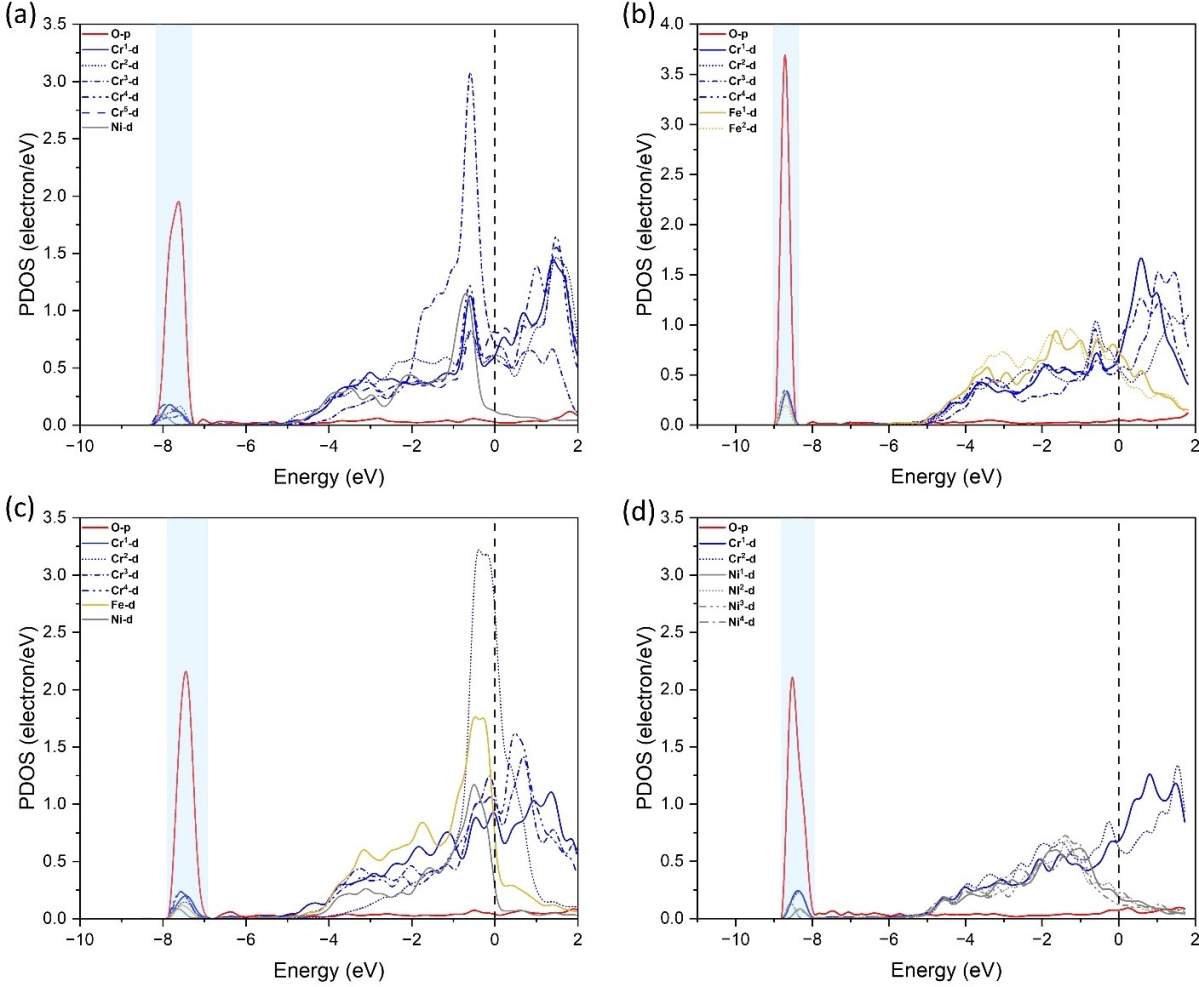
Projected density of states (PDOS) of configurations of FeCrNi (001) surface with one oxygen atom: (a‐b) O at Cr_5_Ni & Fe_2_Cr_4_ and (c‐d) O at FeCr_4_Ni & Cr_2_Ni_4_.

The projected density of states (PDOS) provides information on bonds between different atoms and can be used to assess the stability of different sites. PDOS of oxygen atoms at the adsorption site and subsurface sites is conducted (1^st^ and 2^nd^ subsurface) and is shown in Figure 10 and Figure 11. Here we only consider the valence orbitals *p* for oxygen and *d* for Fe, Cr, and Ni as they take part in bond formation. Figure 10(a–b), Figure 10(c–d), and Figure 10(e–f) are for configurations with the highest ΔFe, ΔCr and ΔNi, respectively. It is observed that oxygen on the surface forms dual peaks with the surrounding metal atoms in the four‐fold at ~−6 eV, while it shifts to a single peak at ~−7.5 eV at the octahedral site in the 1^st^ subsurface. From these PDOS it is evident that oxygen is more stable at the surface rather than the 1^st^ subsurface. Furthermore, this is evident from the adsorption energy and the interstitial energy. In Figure 10(c–d), the energy changes from ~−6 eV to ~−9 eV, and this was the highest energy change associated between the two sites which corresponds to the highest ΔCr for surface to 1^st^ subsurface diffusion.

Fig. 11 shows PDOS at 1^st^ and 2^nd^ subsurface O‐site, where Figure 11(a–b) is for the highest ΔFe and Figure 11(c–d) is for the highest ΔCr and ΔNi. At the 1^st^ and 2^nd^ subsurface O‐sites a single peak is observed at ~−7.5 eV and ~−8.5 eV, respectively. The 2^nd^ subsurface O‐sites have a single peak at lower energy compared to the 1^st^ subsurface O‐site, therefore oxygen is more stable at the 2^nd^ subsurface compared to the 1^st^ subsurface.

## Conclusions

In this paper, we introduce a novel approach to studying interstitial surface diffusion of multi‐principal element alloys, focusing on the principal element effect. The adsorption, absorption, and diffusion of oxygen on FCC FeCrNi MEA were studied by DFT calculations. We started with a randomly arranged FeCrNi supercell, considered the various principal element combinations at the adsorption and stable subsurface O‐sites, and mapped out the energy pathways.

Firstly, oxygen permeation is an endothermic process that starts from a stable adsorption site on the surface and further goes to the nearest O‐site in the subsurface. Cr's positioning at the surface or subsurface is the most important factor for adsorption energy and the permeation barrier.

Secondly, the effect of Fe and Ni on the permeation barrier had less impact on diffusion. A higher concentration of Cr at the surface leads to an increased surface permeation barrier. The 1^st^ subsurface exhibits the highest permeation barrier, leading to low *D* ^*O*^, whereas the permeation barrier decreases for the oxygen atoms from 1^st^ subsurface to the 2^nd^ subsurface, resulting in higher *D* ^*O*^.

Thirdly, it is observed that diffusivity is highly sensitive to ΔCr before and after the jump. Energy barriers and diffusion coefficient suggest that oxidation is affected not only by the principal elements investigated but also by their position on the surface or at the different subsurface. The results of our work can tentatively be used to design oxidation‐resistant surfaces in MEA and HEA for highly corrosive environments and HT applications.

## Code Availability

The Alloy Theoretic Automated Toolkit (ATAT) was used to create the supercell for investigation, it is available free of charge https://www.brown.edu/Departments/Engineering/Labs/avdw/atat/. DFT simulations were performed using the Vienna *Ab initio* Simulation Package (VASP) available for licensing https://www.vasp.at/

## Author Contributions

Farhan Khalid: Writing – original draft, Data curation, Visualization, Methodology, Investigation, Formal analysis. Meifeng Li: Investigation, Writing – review & editing. Jing Liu: Supervision, Project administration, Writing – review and editing, Hao Zhang: Supervision, Funding acquisition, Project administration, Resources, Methodology, Conceptualization, Writing – review & editing.

## Conflict of Interests

The authors declare no conflict of interest.

1

## Supporting information

As a service to our authors and readers, this journal provides supporting information supplied by the authors. Such materials are peer reviewed and may be re‐organized for online delivery, but are not copy‐edited or typeset. Technical support issues arising from supporting information (other than missing files) should be addressed to the authors.

Supporting Information

## Data Availability

The data that support the findings of this study are available from the corresponding author upon reasonable request.
