# Supplementary material for: Oxygen Adsorption, Absorption and Diffusion in FeCrNi Medium Entropy Alloy: An Ab Initio Study
Source: Chemphyschem. 2024 Nov 20;25(24):e202400885. doi: 10.1002/cphc.202400885 (PMC11648836; doi:10.1002/cphc.202400885)
Supplement: Supplementary file 1 — Supporting Information [file CPHC-25-e202400885-s001.pdf]

ChemPhysChem

Supporting Information

# Oxygen Adsorption, Absorption and Diffusion in FeCrNi Medium Entropy Alloy: An Ab Initio Study

Farhan Khalid, Meifeng Li, Jing Liu, and Hao Zhang*

# Supplementary Information

## Oxygen adsorption, absorption and diffusion in FeCrNi medium entropy alloy: an ab initio study

Farhan Khalid, Meifeng Li, Jing Liu and Hao Zhang*

*Department of Chemical and Materials Engineering, University of Alberta, Edmonton, Alberta, T6G, 1H9, Canada.*

* *Corresponding author*, email address: hao.zhang@ualberta.ca

**Table S.1**: Data on interstitial energy of oxygen at octahedral site

| $Fe$ | $Cr$ | $Ni$ | $E_I$ |
|---|---|---|---|
| 0 | 5 | 1 | −2.14 |
| 0 | 2 | 4 | −0.29 |
| 0 | 4 | 2 | −1.37 |
| 1 | 3 | 2 | −1.09 |
| 1 | 1 | 4 | −0.20 |
| 1 | 4 | 1 | −1.72 |
| 1 | 2 | 3 | −0.70 |
| 2 | 1 | 3 | −0.36 |
| 2 | 3 | 1 | −1.33 |
| 2 | 4 | 0 | −1.96 |
| 2 | 2 | 2 | −0.82 |
| 2 | 0 | 4 | 0.44 |
| 3 | 1 | 2 | −0.44 |
| 3 | 3 | 0 | −1.38 |
| 4 | 0 | 2 | −0.09 |
| 4 | 2 | 0 | −1.24 |
| 5 | 0 | 1 | −0.20 |

**Table S.2**: Data on the activation energy for diffusion of oxygen from surface (four-fold hollow site) to 1st subsurface (octahedral site) containing various amount of principal element

| *Start* | | | *End* | | | *Activation energy* | | |
|---|---|---|---|---|---|---|---|---|
| *Fe* | *Cr* | *Ni* | *Fe* | *Cr* | *Ni* | $\Delta H$ | *surface to 1st subsurface* | *1st subsurface to surface* |
| 3 | 1 | 0 | 1 | 4 | 1 | 1.85 | 2.33 | 0.48 |
| 3 | 1 | 0 | 3 | 1 | 2 | 3.12 | 3.12 | 0.00 |
| 3 | 1 | 0 | 4 | 2 | 0 | 2.08 | 2.39 | 0.31 |
| 3 | 1 | 0 | 3 | 3 | 0 | 2.05 | 2.45 | 0.41 |
| 1 | 2 | 1 | 0 | 5 | 1 | 1.39 | 1.50 | 0.11 |
| 1 | 2 | 1 | 0 | 2 | 4 | 3.53 | 3.53 | 0.00 |
| 1 | 2 | 1 | 2 | 3 | 1 | 2.38 | 2.58 | 0.21 |
| 1 | 2 | 1 | 0 | 4 | 2 | 2.50 | 2.50 | 0.00 |
| 0 | 3 | 1 | 1 | 4 | 1 | 2.27 | 2.27 | 0.00 |
| 0 | 3 | 1 | 2 | 4 | 0 | 2.31 | 2.31 | 0.00 |
| 0 | 3 | 1 | 2 | 2 | 2 | 3.25 | 3.25 | 0.00 |
| 1 | 3 | 0 | 3 | 1 | 2 | 3.56 | 3.56 | 0.00 |
| 1 | 3 | 0 | 3 | 3 | 0 | 2.63 | 3.75 | 1.12 |
| 2 | 1 | 1 | 3 | 1 | 2 | 2.85 | 3.37 | 0.52 |
| 2 | 1 | 1 | 1 | 4 | 1 | 1.55 | 1.55 | 0.00 |

**Table S.3**: Data on the activation energy for diffusion of oxygen from 1st subsurface (octahedral site) to 2nd subsurface (octahedral site) containing various amount of principal element

| *Start* | | | *End* | | | *Activation energy* | | |
|---|---|---|---|---|---|---|---|---|
| *Fe* | *Cr* | *Ni* | *Fe* | *Cr* | *Ni* | $\Delta H$ | $1^{st}$ *to* $2^{nd}$ | $2^{nd}$ *to* $1^{st}$ |
| 1 | 4 | 1 | 0 | 2 | 4 | 1.27 | 1.27 | 0.00 |
| 1 | 4 | 1 | 0 | 5 | 1 | −0.27 | 0.78 | 1.05 |
| 4 | 2 | 0 | 1 | 3 | 2 | 0.27 | 0.76 | 0.49 |
| 4 | 2 | 0 | 2 | 3 | 1 | 0.11 | 0.74 | 0.63 |
| 3 | 3 | 0 | 0 | 5 | 1 | −0.47 | 0.78 | 1.24 |
| 3 | 3 | 0 | 1 | 3 | 2 | 0.21 | 0.78 | 0.57 |
| 2 | 3 | 1 | 1 | 2 | 3 | 0.39 | 0.69 | 0.29 |
| 2 | 3 | 1 | 2 | 4 | 0 | −0.29 | 0.69 | 0.97 |
| 2 | 3 | 1 | 1 | 4 | 1 | −0.52 | 0.77 | 1.29 |
| 0 | 5 | 1 | 2 | 4 | 0 | 0.74 | 1.44 | 0.70 |
| 0 | 5 | 1 | 1 | 4 | 1 | 0.47 | 2.45 | 1.99 |
| 0 | 5 | 1 | 2 | 2 | 2 | 1.23 | 1.73 | 0.50 |
| 3 | 1 | 2 | 4 | 2 | 0 | −0.06 | 1.42 | 1.49 |
| 3 | 1 | 2 | 1 | 1 | 4 | 0.19 | 0.46 | 0.27 |
| 3 | 1 | 2 | 2 | 2 | 2 | −0.88 | 0.21 | 1.09 |
| 1 | 3 | 2 | 2 | 3 | 1 | 0.55 | 1.16 | 0.62 |

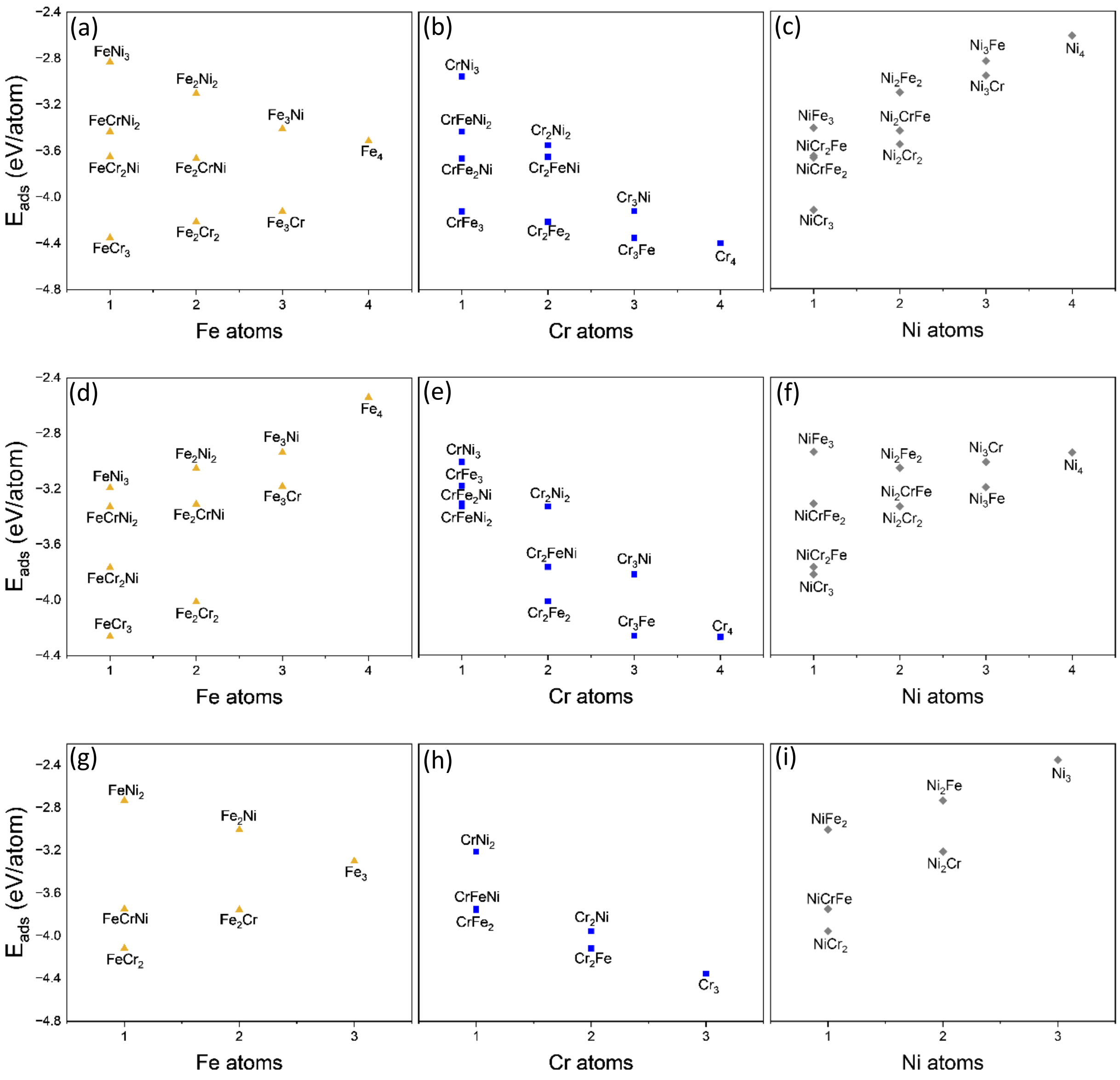


**Figure S.1**: The adsorption energy of oxygen atom at the adsorption site surface versus the type of its neighboring atoms. (a-c) shows the four-fold site adsorption energy for the (001) surface, (d-f) shows the four-fold site adsorption energy for the (110) surface, and (g-i) shows the three-fold site adsorption energy for the (111) surface. The x-axis shows the increasing principal atom.
